# Adhesions After Laparoscopic IPOM—How Serious Is the Problem?

**DOI:** 10.3389/jaws.2025.14126

**Published:** 2025-03-26

**Authors:** Henry Hoffmann, Philipp Kirchhoff

**Affiliations:** ^1^ ZweiChirurgen GmbH, Center for Hernia Surgery and Proctology, Basel, Switzerland; ^2^ Faculty of Medicine, University Basel, Basel, Switzerland; ^3^ Merian Iselin Clinic, Clinic for Orthopedics and Surgery, Basel, Switzerland

**Keywords:** laparoscopic IPOM, ventral hernia, adhesions, laparoscopic IPOM+, hernia

## Abstract

Although laparoscopic IPOM is still the gold standard in ventral hernia repair, it is subject of a slow but constant decline, while new minimally invasive techniques are increasingly used, as well as open retromuscular repairs. One of the reasons are the intraperitoneal mesh position and its suspected higher risk for creating intraabdominal adhesions, compared to extraperitoneal mesh positions. In potential subsequent operations (e.g., in recurrent ventral hernia repair) adhesions usually must be taken down, which is a known risk factor for complications such as inadvertent enterotomies, surgical site infections and prolonged hospital stay. In this review we evaluate the incidence of intraabdominal adhesions after ventral hernia repair and their potential impact on surgical outcome in subsequent operations. Special attention is paid to the impact of mesh position in developing adhesions.

## Introduction

Laparoscopic repair of ventral or incisional hernias with intraperitoneal mesh (laparoscopic IPOM) is still the gold standard of care, with low rates of surgical and general postoperative complications as well as complication-related reoperations [1, 2].

Many factors are thought to influence the postoperative outcome of laparoscopic IPOM [2]. More recently the intraperitoneal mesh and its potential effects are in the focus of hernia surgeons worldwide. The main concern roots from the intraperitoneal mesh position and its potential adverse events. Subsequentially, the laparoscopic IPOM seems to be on a decline, which has been shown in a recent large hernia registry analysis [3]. In contrast, alternative techniques such as minimally-invasive extraperitoneal repairs eTEP (extended totally extraperitoneal plasty or total extraperitoneal repair) or MILOS (Mini Less Open Surgery) are on a rise [3–5]. They show advantages over laparoscopic IPOM and open mesh repair [4] with significantly lower rates of mesh-related complications, bowel obstructions, mesh infections, fistulae, and mesh-related reoperations in the 5-year follow-up. Surprisingly, open retromuscular repairs are increasingly used as well [3], although the disadvantages of this approach especially regarding wound complications are well documented [4].

There are well documented surgeon-related [6] and patient-related factors [7–9] regarding the postoperative outcome in laparoscopic IPOM. Also, mesh-related problems have been described, which may have rooted from a design flaw [10–13]. In addition, mesh features including textile and structural components seem to play an important role for postoperative outcomes, such as recurrence and mesh-related complications. Under ideal circumstances the mesh should combine antimicrobial features, excellent tissue integration to the abdominal wall, long-lasting and strong mechanical performance, low rate of visceral adhesion and minimal inflammatory response or foreign body reactions [14]. The only approved preventive mesh features against adhesions are anti-adhesive barriers, such as implanted hydrogels, that physically separate internal tissues following surgery. Although gel-coated meshes with antiadhesive barriers are routinely used for laparoscopic IPOM repairs [14], data show that the clinical use of hydrogels has not yet significantly reduced the incidence of adhesion-related disease, challenging the long-lasting effect of anti-adhesive barriers [15]. Mesh features seem to be of importance, since the risk of re-operation with potential adhesiolysis is affected by type of mesh too [16]. Therefore, further developments in mesh technology a needed to improve the composite membranes and other features to reliably preventing intraabdominal adhesions [17].

In this review article we want to evaluate the incidence of mesh-related adhesions after laparoscopic IPOM, the process of adhesions development, the symptoms they can create and what clinical impact they may have.

## How Often Will Adhesions Develop After Abdominal Surgery and Ventral Hernia Repair?

Intraabdominal adhesions are an inevitable consequence of any abdominal surgery. Most patients who undergo subsequent abdominal surgery will present adhesions from previous operations and require adhesiolysis [18, 19] in most cases. The reported incidence of intraperitoneal adhesions after general abdominal operations ranges from 67%–93% [20, 21]. Generally, laparoscopic operations carried the lowest risk for forming adhesions [22]. To prevent adhesions careful tissue dissection and minimal trauma during surgery seems of utmost importance to reduce the risk of adhesions, which includes limiting tissue damage and the amount of foreign material, such as sutures, drains and meshes [23].

For most patients, adhesions are of no adverse consequence, but to some they are troublesome. Approximately one-third of the patients undergoing intraabdominal surgery are later readmitted to the hospital for problems possibly related to these adhesions with rates of adhesion-related re-operations in up to 5% [24, 25]. Especially major abdominal operations carry a high risk for adhesion-related complications after the surgery. For example, the 5-year hospitalization risks for bowel obstruction due to adhesion-related complications are reported to range between 11% and 25% [22]. Band adhesions are the most common cause of intestinal obstruction, with as many as 20% of patients readmitted with symptoms suggestive of obstruction at some point following major abdominal surgery [26, 27]. When operating on this patient group, adhesions will likely cause lengthy, time-consuming, and potentially dangerous tissue and bowel dissection, in which inadvertent enterotomy occurs in at least 5% [27]. Therefore, post-surgical peritoneal adhesions are a major health burden for patients and healthcare providers, accounting for over 300,000 additional abdominal operations per year in the United States of America with annual costs of several billion dollars [28].

When it comes to hernia surgery, especially IPOM meshes are under suspicion of causing extensive adhesions, which has led to a downward trend in the use of laparoscopic IPOM in the last decade [3]. It must be stated that adhesions form in the majority of laparoscopic IPOM meshes despite their anti-adhesive barrier [29, 30]. In one register study it was demonstrated that patients having undergone laparoscopic IPOM have an increased risk of bowel obstruction compared with patients who have a similar surgical history but no laparoscopic IPOM repair [31]. In a series of re-do laparoscopies following laparoscopic IPOM 42% of patients had omental and 11% had serosal bowel adhesions to the mesh [32]. The degree and extend of adhesions after laparoscopic IPOM show an association of the unique properties of the mesh and the anti-adhesive barrier [33]. For example, when polypropylene mesh with omental interposition was used, 35% of patients showed detectable adhesions during ultrasound examinations [34]. In contrast, mild and moderate adhesions where seen in 83% of cases during re-operations in patients who underwent laparoscopic IPOM with anti-adhesive barriers [35]. There are also studies comparing different IPOM meshes and their potential difference in adhesion-related complications such as postoperative ileus [11–13]. There have been differences reported, but the main limitation of some studies is their retrospective character, with low generalizability for clinical practice [12, 13]. One prospective comparative study could not demonstrate significant differences between the compared meshes [11].

Recurrence and potential reoperation are inevitable consequences after ventral hernia repair showing only minor differences between open, laparoscopic and robotic-assisted techniques [36, 37]. Also, adhesions related complications seem to appear in all kinds of mesh position (retromuscular, preperitoneal and intraperitoneal) in ventral hernia repair. For example, the 5-year hospitalization risks for bowel obstruction thought to be adhesion-related are reported to be up to 14% after abdominal wall hernia surgery [22, 38]. There is also data suggesting advantages of laparoscopic IPOM compared to open retromuscular repair. A long-term prospective register study of the Danish Hernia database, which included 3,242 elective incisional hernia operations from 2007 to 2010 with a median follow-up period of 60 months and 100% follow-up rate, reported mesh-related complication rates of 5.6% after open mesh and 3.7% after laparoscopic mesh repair [39], questioning the fear of higher adhesions in IPOM meshes. It could be shown that mesh-related surgical complication after the index hernia repair appear significantly earlier in the open mesh group compared to the laparoscopic IPOM group (11 vs. 24 months). On the other hand, life-threatening complications occurred in 0.9% of patients with open mesh repair and 1.8% of patients with laparoscopic mesh repair, without reaching a level of statistical significance. Open mesh repair was shown to be a risk factor *per se* for long-term complications in a propensity-adjusted analysis (HR 2.36, p > 0.01), showing more patients requiring re-operations due to mesh-complications in the open repair group compared to the laparoscopic IPOM group. Re-do operation as such and not particularly IPOM meshes seem to be the major reason for higher rates for intraoperative, postoperative and general complications in recurrent incisional hernia repair [37, 40, 41]. A study evaluating American healthcare data from Medicare including 85′663 patients with a 5-year follow-up showed that initial major abdominal operations (25% vs. 8%, p < 0.001) and incisional hernia repairs (12% vs. 1%, p < 0.001) carried the highest risk for adhesion-related complications compared to their control groups [22]. This data questions the role of the mesh and its position as a risk factor for developing adhesions too.

## What Are the Reasons for Developing Adhesions

The development of intraperitoneal adhesion has not yet been fully understood but is associated with patient-related, mesh-related, procedure-related and molecular-level factors [42]. The critical period when most adhesions form is the first week after implantation of the mesh [43]. It cases of laparoscopic IPOM it has been shown that larger meshes and higher Charlson morbidity index of the patients are independent predictors for developing adhesions [44]. Also, it could be shown in animal studies that higher CO^2^ insufflation pressure in laparoscopic surgery and peritoneal desiccation seem to promote more adhesions [45]. On the cellular and molecular level the process of developing adhesions contains a complex cascade of inflammatory processes suppressing fibrinolytic activity [46]. For example, intraoperative contamination of gut microbes increases the risk of post-surgical adhesion formation. This transformation is driven by epidermal growth factor receptor (EGFR) signaling [47]. The post-surgical adhesions form when two mesothelial surfaces are attached to each other by connective tissue by a fibrotic reaction. This process can be initiated by coagulation, aggregation of macrophages, and intercellular adhesions between mesothelial cells [48–50].

## Diagnostic Imaging and Patterns of Adhesions

Besides detailed medial history taking and profound abdominal examination there are several potential imaging-based diagnostic tools including ultrasound and magnetic resonance imaging (MRI) [44, 51–53]. MRI can detect adhesions between bowel and abdominal wall in a reliable way, showing that adhesions are formed both after open and laparoscopic hernia mesh repair [44]. MRI evaluations revealed a certain pattern on how adhesions form in the abdominal cavity. Adhesion formation between bowel and abdominal wall after ventral hernia repair with mesh seem to form more often in der periumbilical area and the lower mid abdomen in the suprapubic region [44]. Especially small bowel seems to be at risk for forming adhesions, while colon almost never seems to form adhesions to the mesh or the abdominal wall [44].

## Intraperitoneal Adhesions and Chronic Abdominal Pain

Besides intestinal obstruction due to band adhesions which require treatment including adhesiolysis [26, 27] one of the assumed problems of intraabdominal adhesion is chronic abdominal pain, which has been reported with a prevalence of up to 40% after general abdominal operations [54]. In older pathology studies is was revealed that adhesions contain nerve fibers and may itself cause pain [48, 55, 56], and adhesions can stimulate stretch receptors in the smooth muscle of the abdominal wall and the bowel [23]. However, this hypothesis has never been proven in more recent studies. In addition, adhesions cause intraabdominal fibrotic scarring, resulting in restricted organ movement, and potential bowel obstruction and infertility, creating a significant economic burden [25, 57]. Awake laparoscopy revealed that filmy adhesions between a movable structure, such as an ovary and the peritoneum had the highest pain scores. Fixed or dense adhesions, no matter where they were located, had the lowest pain scores [58]. However, although there are several known risk factors for chronic postoperative abdominal pain such as patient characteristics, psychological factors, procedure-related factors and pre- and acute postoperative pain [54], there is no strong evidence that intraabdominal adhesions cause pain. This could be demonstrated by a large review of 196 papers [27], showing no clear evidence that chronic abdominal pain and intraabdominal adhesions are linked to each other. This is supported by MRI studies suggesting that adhesions form both after open and laparoscopic hernia mesh repair and are not associated with chronic pain [44]. Therefore, it remains a matter of debate if laparoscopic adhesiolysis is a sufficient treatment option in patients with chronic abdominal pain. In a double blinded randomized controlled trial comparing laparoscopy alone vs laparoscopic adhesiolysis in patients with chronic abdominal pain both treatment groups showed substantial pain relief and better QoL after the intervention [59]. A 12 years follow up revealed that laparoscopic adhesiolysis was less beneficial than laparoscopy alone in the long term. This emphasizes, that there appears to be a powerful, long-lasting placebo effect of laparoscopy alone, which may be explained by the fast that additional adhesiolysis is associated with an increased risk of intraoperative complications. In summary, avoiding adhesiolysis in diagnostic laparoscopy may result in less morbidity and healthcare costs with better results on the long run [60].

## Risk of Inadvertent Enterotomy in Reoperations

Inadvertent enterotomies as one of the most serious intraoperative complication occur in up to 2% of patients undergoing primary laparoscopic or robotic ventral hernia repair [61, 62]. Patients suffering from inadvertent enterotomy will be faced with a longer length of stay, higher healthcare cost, more infections, readmissions, re-operations and higher mortality rates [63].

In the last decade laparoscopic IPOM is subject of a clear decline [3]. This trend may be explained by the fact that surgeons fear intraabdominal adhesions after laparoscopic IPOM and complications which may be associated with an intraabdominal foreign material, especially during subsequent abdominal operations [63]. This apprehension is driven by the fact, that adhesiolysis during abdominal surgery can cause iatrogenic organ injury (inadvertent enterotomy), increased operative time and a prolonged recovery period, showing significantly lower pre- and postoperative functional status measured by SF-36 and DASI Score (p < 0.01) [64]. In an analysis of the Swedish National Patient Register between 2010 and 2019 including 29,360 umbilical and 6,514 epigastric hernia repairs, the risk of re-operation after umbilical hernia repair was significantly lower in open interstitial and open sublay repair compared to suture repair. In contrast, laparoscopic and open IPOM repairs had the same risk for re-operation compared to suture repair [37], showing a disadvantage of laparoscopic IPOM compared to interstitial and retromuscular mesh repairs. This trend however could not be demonstrated for epigastric hernia repair. Here, all repair techniques showed no difference regarding the risk of re-operation [37].

Considering the fact that all ventral hernia repairs can cause adhesions and lead to potential bowel obstruction after surgery [65], it is not surprising that reviewing the available data regarding potential risk of inadvertent enterotomy during re-do surgery after laparoscopic IPOM reveals contradictory study results. In one study it could be demonstrated that the rates of inadvertent enterotomies and unplanned bowel resection increases significantly in cases of recurrent hernia repair with previous mesh [66]. Another study revealed that bowel obstruction happens more often after laparoscopic IPOM compared to extraperitoneal mesh position, but this difference was found not to be statistically significant [65]. In contrast, the risk for developing adhesions especially after laparoscopic IPOM has been reported to be lower after laparoscopic compared to open hernia surgery [46].

## Discussion

Despite being the gold standard in ventral hernia repair laparoscopic IPOM technique is on a decline compared to open sublay repairs and new minimally invasive extraperitoneal techniques. One of the reasons may be the critical view of many surgeons on the intraperitoneal mesh position and the assumed risk of higher mesh-related complications. Although IPOM meshes are featured with anti-adhesive barriers, most of them will develop adhesions. Numerous animal studies have revealed differences of adhesion formations between different IPOM meshes. However, these results have never been followed-up in studies evaluating human patients. While adhesions do not seem to be linked to chronic abdominal pain after surgery, they carry the risk for bowel obstruction and inadvertent enterotomy during subsequent surgery. However, available data are contradictory if intraperitoneal mesh position is a risk factor for adhesions. It can be stated that all mesh positions can cause adhesions. In summary, the available data cannot support nor refute the concerns of higher rates of adhesions and adhesion-related complications after ventral hernia surgery with intraperitoneal meshes (IPOM).
